# Suppression of histone deacetylation promotes the differentiation of human pluripotent stem cells towards neural progenitor cells

**DOI:** 10.1186/s12915-014-0095-z

**Published:** 2014-11-19

**Authors:** Juan Yang, Yu Tang, Hui Liu, Fang Guo, Jun Ni, Weidong Le

**Affiliations:** Key Laboratory of Stem Cell Biology, Institute of Health Sciences, Shanghai Institutes for Biological Sciences, Chinese Academy of Sciences/Shanghai Jiao Tong University School of Medicine, Shanghai, China; Center for Translational Research of Neurology Disease, The 1st Affiliated Hospital, Dalian Medical University, Dalian, China

**Keywords:** Histone deacetylation, Histone deacetylase inhibitors, Neuroectodermal specification, Neural progenitor cells, Human pluripotent stem cells

## Abstract

**Background:**

Emerging studies of human pluripotent stem cells (hPSCs) raise new prospects for neurodegenerative disease modeling and cell replacement therapies. Therefore, understanding the mechanisms underlying the commitment of neural progenitor cells (NPCs) is important for the application of hPSCs in neurodegenerative disease therapies. It has been reported that epigenetic modifications of histones play important roles in neural differentiation, but the exact mechanisms in regulating hPSC differentiation towards NPCs are not fully elucidated.

**Results:**

We demonstrated that suppression of histone deacetylases (HDACs) promoted the differentiation of hPSCs towards NPCs. Application of HDAC inhibitors (HDACi) increased the expression of neuroectodermal markers and enhanced the neuroectodermal specification once neural differentiation was initiated, thereby leading to more NPC generation. Similarly, the transcriptome analysis showed that HDACi increased the expression levels of ectodermal markers and triggered the NPC differentiation related pathways, while decreasing the expression levels of endodermal and mesodermal markers. Furthermore, we documented that HDAC3 but not HDAC1 or HDAC2 was the critical regulator participating in NPC differentiation, and knockdown of HDAC3′s cofactor SMRT exhibited a similar effect as HDAC3 on NPC generation.

**Conclusions:**

Our study reveals that HDACs, especially HDAC3, negatively regulate the differentiation of hPSCs towards NPCs at an earlier stage of neural differentiation. Moreover, HDAC3 might function by forming a repressor complex with its cofactor SMRT during this process. Thus, our findings uncover an important epigenetic mechanism of HDAC3 in the differentiation of hPSCs towards NPCs.

**Electronic supplementary material:**

The online version of this article (doi:10.1186/s12915-014-0095-z) contains supplementary material, which is available to authorized users.

## Background

Human pluripotent stem cells (hPSCs) including human embryonic stem cells (hESCs) and human induced pluripotent stem cells (hiPSCs) possess the potential to differentiate into all cell types of three germ layers, which raises new prospects for disease modeling, molecular mechanism finding and cell-replacement therapies of neurodegenerative diseases that have a very complex nature of pathogenesis involving both neurons and glial cells [1-4]. The major challenge is how to efficiently generate renewable neural progenitor cells (NPCs), which can be further differentiated into neurons, astrocytes or oligodendrocytes under different conditions, for the study of cell-autonomous or non-cell-autonomous effects on disease development and cell replacement therapy [5,6]. Therefore, exploring the mechanisms underlying NPC commitment during neural differentiation will be of great importance.

It has been reported that multiple signaling pathways are involved in neural induction from hPSCs. The retinoic acid (RA) signaling pathway works synergistically with sonic hedgehog (SHH) and bone morphogenetic proteins (BMPs) as well as fibroblast growth factors (FGFs) signaling pathways to determine the extending and dorso-ventral patterning of neural tubes during early embryonic development [7]. RA is a critical morphogen for the generation of NPCs [8,9]. In addition, specification of ectoderm into neural cells and epidermal cells is directed by Wnt, BMP and FGF pathways [10-12]. Blocking the BMP/SMAD signaling pathway with small molecules up-regulates the expression of neural transcription factors, such as SOX2, PAX6 and NESTIN, and, therefore, facilitates NPC generation from hPSCs [13]. Moreover, FGF2 can antagonize BMP signaling to stabilize neural identity during early neural specification [14].

Generally, neural differentiation of hPSCs consists of two major processes: NPC generation from hPSCs and final neuronal or glial conversion from the NPCs. Epigenetic modifications of DNA and histones play important roles in the transcriptional regulation of neural cell fate-specific genes [15,16]. Among those epigenetic processes, histone deacetylases (HDACs) participate in neural differentiation by modifying histone acetylation/deacetylation levels. It has been reported that HDAC inhibitors (HDACi) such as valproic acid (VPA), sodium butyrate (NaB) and trichostatin A (TSA) can promote neuronal differentiation of NSCs/NPCs isolated from adult mouse brain, while suppressing their differentiation into oligodendrocytes and astrocytes [17-19]. However, there is a somewhat controversial report showing that deletion of the histone acetyltransferase (HAT) cofactor transformation/transcription domain-associated protein (*Trrap*) can improve the differentiation of NPCs into neurons in mice [20], implying that disruption of the HAT recruitment can also enhance neuronal differentiation. Thus, the mechanisms by which histone acetylation or deacetylation affects neural differentiation might be complicated. Moreover, those studies mainly demonstrate that the process of neuron or glial cell specification from NPCs, which is the latter process of neural differentiation, can be modulated by HDACs. However, the potential roles of HDACs in the early stage of neural differentiation in mediating hPSC differentiation towards NPCs have yet to be determined.

Here we report that suppression of HDACs remarkably enhances the generation of NPCs. We find that HDACi, such as NaB and mocetinostat (MGCD0103, MGCD), can promote neuroectodermal specification once the neural differentiation is initiated. Furthermore, we document that HDAC3 might negatively regulate NPC commitment by forming a repressor complex with its cofactor SMRT (also known as nuclear receptor corepressor 2, NCOR2); knocking down of HDAC3 or SMRT in hPSCs exhibits similar effects on the generation of NPCs. Collectively, our studies uncover an important epigenetic mechanism of HDAC3 modification in the differentiation of hPSCs towards NPCs, which may facilitate the establishment of an optimized strategy of NPC generation from hPSCs for basic science and application research.

## Results

### HDACi promote the generation of NPCs

**Figure 1:**
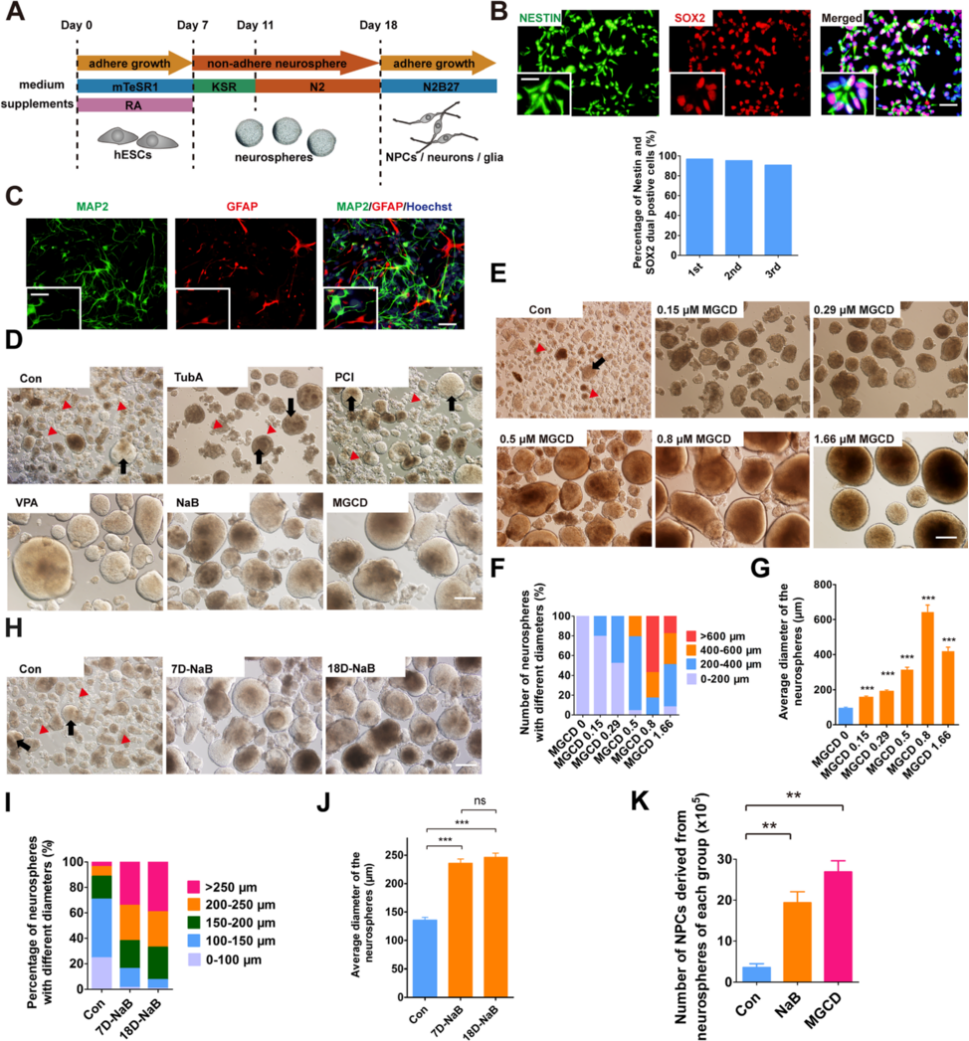
**HDACi greatly enhance hPSCs differentiation towards NPCs. (A)** Schematic representation of the neural differentiation in monolayer culture. **(B)** Immunostaining of cells derived from neurospheres with antibodies against NESTIN (green) and SOX2 (red) and quantification of NESTIN and SOX2 dual positive cells on day 18 of differentiation. The nuclei were stained by Hoechst. Scale bar, 50 μm. The inset shows a higher magnification view. Scale bar, 100 μm. **(C)** Immunostaining of cells after 40 days of spontaneous differentiation with antibodies against MAP2 (green) and GFAP (red). The nuclei were stained by Hoechst. Scale bar, 50 μm. The inset shows a higher magnification view. Scale bar, 100 μm. **(D)** Neurospheres were formed after HDACi treatment for seven days. The sizes of neurospheres were analyzed on day 18 of differentiation. Black arrows represent typical neurospheres, and red arrowheads represent cell debris. **(E-G)** Neurospheres were formed after MGCD treatment for seven days and the diameters of the neurospheres were measured and quantified on day 18, respectively. **(H-J)** Neurospheres were formed after NaB treatment for seven days or eighteen days and the diameters of the neurospheres were detected and quantified on day 18 of differentiation, respectively. Scale bars, 200 μm. The error bars indicate SEM; ns, not significant; ***P <0.001; n >50. **(K)** The efficiency of NPC generation was assessed by counting the number of cells derived from digested neurospheres on day 18. **P <0.01; n >/=3. HDACi, HDAC inhibitors; hPSCs, human pluripotent stem cells; NPC, neural progenitor cell; SEM, standard error of the mean.

The H9 hESC line used in our study was in an undifferentiated state. This cell line expressed pluripotent markers SOX2 and OCT4 but not NPC markers NESTIN or PAX6 (see Additional file 1: Figure S1A). We initiated the neural induction of H9 by RA treatment, followed by neurosphere formation (Figure 1A). We found that many cells died at the beginning of neurosphere formation. However, nearly 95% of cells derived from neurospheres were NESTIN and SOX2 dual positive NPCs at the end of this stage (Figure 1B), suggesting that the cells which finally contributed to NPC generation survived during the neurosphere formation. Moreover, the NPCs could spontaneously differentiate into neurons and glial cells in NPC medium without bFGF (Figure 1C). These results suggest that typical NPCs with integrated differentiation potentials are generated using this strategy. Therefore, we utilized this model of neural differentiation to study the mechanisms underlying the differentiation of hPSCs towards NPCs.

It is reported that retinoic acid receptors (RARs) recruit HDACs to repress differentiation related genes [21], indicating that histone deacetylation can regulate the RA signaling pathway. Our results showed that transcripts of class I and class II HDACs were increased once the differentiation was initiated and were kept at high levels in all stages, indicating that HDACs participate in the process of neural differentiation (see Additional file 1: Figure S1B). To investigate whether HDACs play roles in NPC generation, we suppressed histone deacetylation using several HDACi. Of those, VPA (from 0.3 mM to 3 mM) and NaB (from 50 μM to 1 mM) are pan-inhibitors of class I and class II HDACs [17,22,23]. First, we treated H9 cells with NaB and VPA at various concentrations for 24 hours to evaluate the toxicity of those HDACi. We found that most H9 cells changed their morphology and died when treated with NaB at 0.5 mM to 5 mM or with VPA at 1 mM to 5 mM (see Additional file 2: Figure S2A). Therefore, we chose 0.1 mM of NaB and 0.5 mM of VPA for our study. We also applied tubastatin A (TubA) and PCI-34051 (PCI) at 5 μM, and MGCD at 0.5 μM, the concentrations of which were shown to inhibit HDAC6 or HDAC8 or HDAC1-3 without obvious cytotoxicity [24,25].

We then treated H9 cells with those HDACi with optimal concentrations at the stage of RA induction. Interestingly, we found that the number and size of the neurospheres were significantly increased upon VPA, NaB or MGCD treatment. However, we did not observe significant changes in the PCI- or TubA-treated cells compared to the untreated cells (Figure 1D), suggesting that class I HDACs, especially HDAC1, HDAC2 and HDAC3, may play more important roles than other HDACs in NPC generation.

As MGCD inhibited HDAC1, HDAC2 and HDAC3 in a dose-dependent manner, we then tested the effects of MGCD at different concentrations (Figure 1E), showing that the neurospheres became larger as MGCD concentration increased and about 90% of the neurospheres were larger than 200 μm in diameter when MGCD was used at 0.5 μM to 1.66 μM (Figure 1F and G).

To explore whether HDACi treatment affected the growth of neurospheres, we continuously treated the neurospheres with NaB at the second stage in which the neurospheres were formed and grown (Figure 1H). We observed that the neurospheres from 18-day-treatment cells (18D-NaB) were comparable in size and number to those from 7-day-treatment cells (7D-NaB). More than 70% of the neurospheres from both treatments were larger than 200 μm, while more than 40% of the neurospheres from control cells were smaller than 150 μm (Figure 1I). Moreover, the average diameters of neurospheres from 7D-NaB and 18D-NaB showed no significant difference (Figure 1J), suggesting that NaB treatment at the second stage does not change the growth of neurospheres and persistent HDACi treatment is unnecessary. In other words, HDACi may play a role at the stage of RA induction but not at the stage of neurosphere formation. In fact, we documented that incubating with MGCD for only three days was sufficient to enhance the NPC generation (see Additional file 2: Figure S2B), suggesting that HDACi might promote the NPC commitment at a very early time point of the NPC differentiation.

Importantly, as the above neurosphere morphology changes directly reflect the efficiency of NPC generation, we determined the efficiency by calculating the number of cells derived from neurospheres on day 18. The neural differentiation of hPSCs with or without HDACi treatment was initiated in 6-cm dishes with the same cell number. At the end of the second stage, we found that the number of NPCs derived from the neurospheres of both NaB- and MGCD-treated cells was 10- to 15-fold more than that of the control cells (Figure 1K). These results indicate that suppression of HDACs by HDACi can significantly enhance the differentiation of NPCs from hESCs.

### HDACi do not alter the properties of NPCs

**Figure 2:**
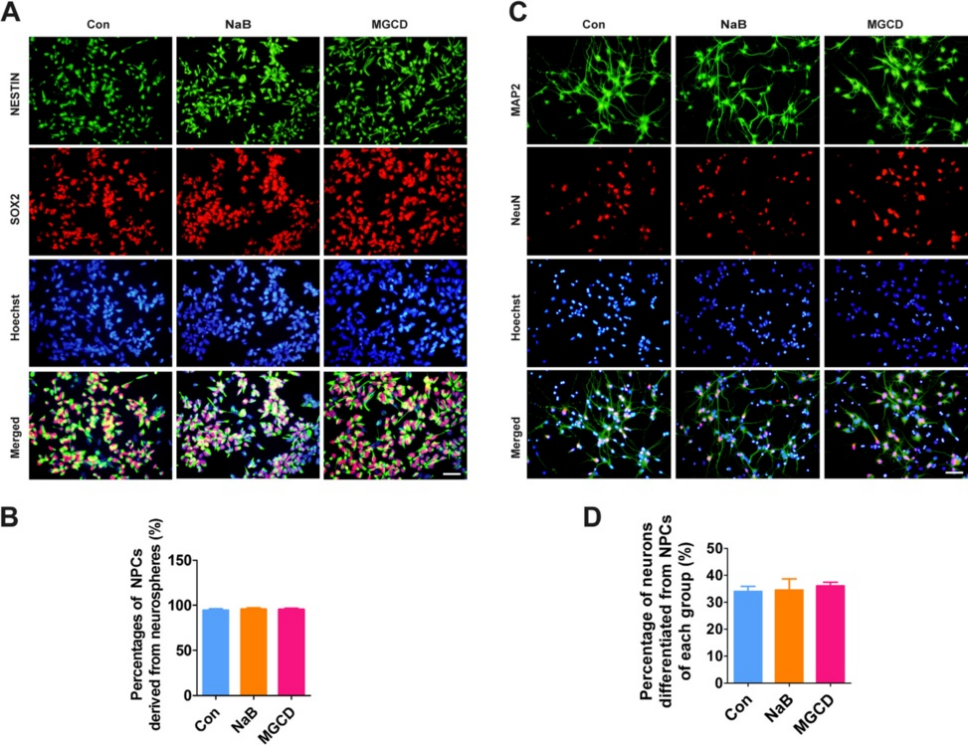
**HDACi do not alter the properties of generated NPCs. (A)** Neurospheres were digested and plated on dishes. NPCs were then visualized using immunostaining with anti-NESTIN (green) and anti-SOX2 (red) antibodies. **(B)** The percentages of NPCs were calculated, the error bars indicate SEM; n = 4. **(C,** **D)** NPCs were differentiated into neurons, which was detected by immunostaining with anti-MAP2 (green) and anti-NeuN (red) antibodies after 50 days of differentiation. The efficiency of neuronal differentiation was compared among control cells and HDACi treated cells. Scale bars, 50 μm. The error bars indicate SEM; n>/=3. HDACi, HDAC inhibitors; NPCs, neural progenitor cells; SEM, standard error of the mean.

We then attempted to identify whether the properties of generated NPCs were influenced by the HDACi treatment. We showed that about 95% of the neurosphere cells from HDACi-treated cells were typical NPCs as detected by NESTIN and SOX2 immunostaining, manifesting no significant differences compared with control cells (Figure 2A and B). Furthermore, we found that NPCs could differentiate into mature neurons expressing MAP2 and NeuN with comparable efficiencies (Figure 2C and D), demonstrating that HDACi do not compromise the neuronal differentiation potential of the generated NPCs. Taken together, these results indicate that HDACs function at the early stage of NPC differentiation from hPSCs without affecting the integrity of neuronal differentiation of the NPCs.

### HDACi promote neuroectodermal specification

**Figure 3:**
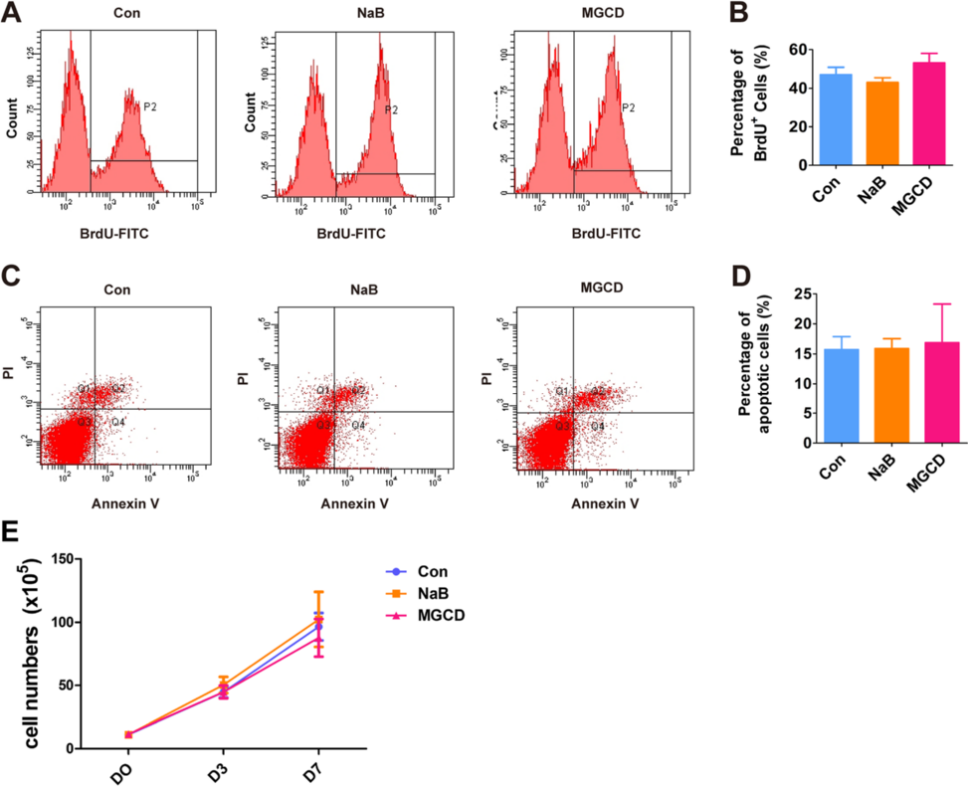
**Proliferation and apoptosis analysis. (A, B)** The proliferation ability of H9 cells after HDACi treatment on day 3 of differentiation was assessed by BrdU incorporation. The error bars indicate SEM; n>/=4. **(C, D)** The apoptosis of H9 cells after HDACi treatment on day 3 of differentiation was detected by Annexin V/PI staining. The error bars indicate SEM, n = 4. **(E)** The cell number of H9 cells during differentiation after HDACi treatment was counted. The error bars indicate SEM; n = 4. BrdU, bromodeoxyuridine; HDACi, HDAC inhibitors; SEM, standard error of the mean.

In order to explore the mechanisms of HDACs in NPC generation, we focused on the first stage of differentiation when hPSCs committed to different cell lineages. We found that NaB and MGCD altered neither the proliferation of cells (Figure 3A and B) nor cell survival (Figure 3C and D). The cell number of HDACi-treated cells was similar to that of untreated cells during differentiation (Figure 3E), suggesting that cell growth during differentiation is not affected by HDACi treatment.

**Figure 4:**
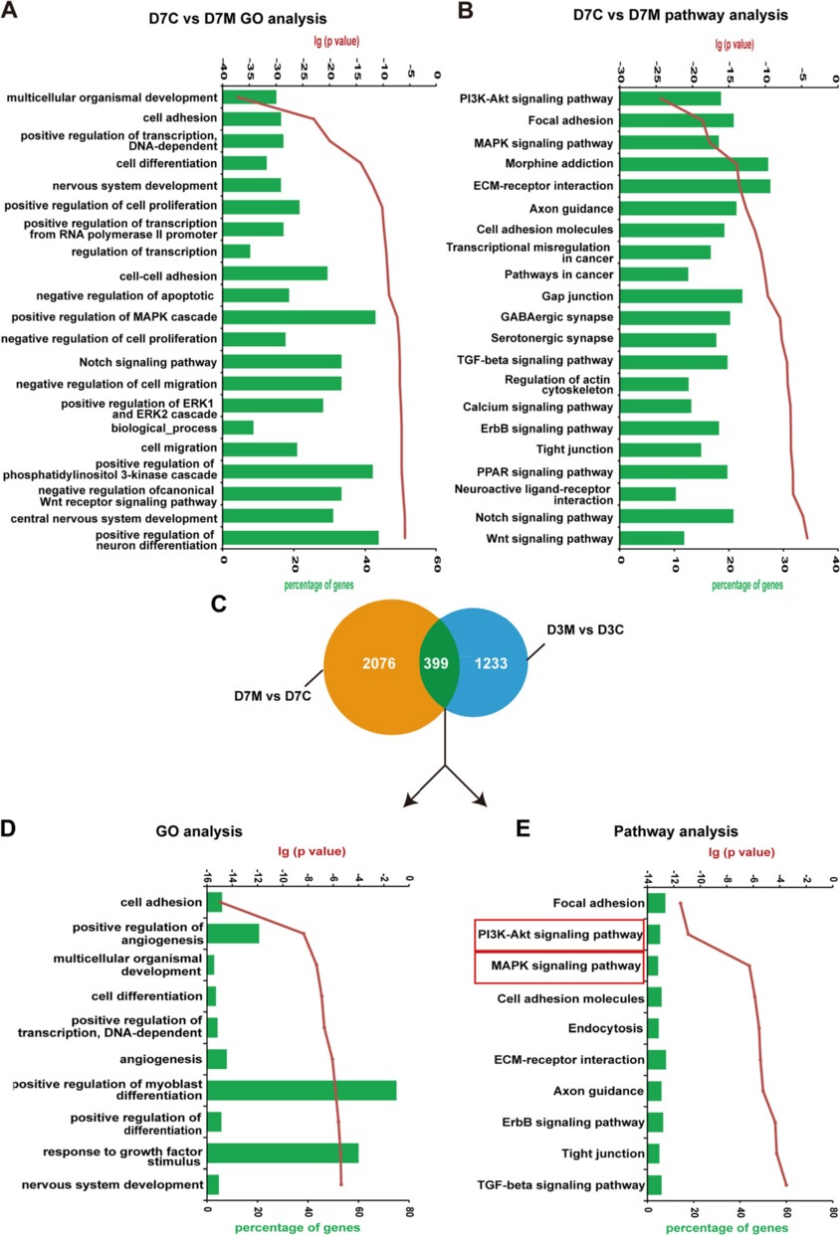
**Gene-expression patterns and GO analysis in H9 cells with or without MGCD treatment. (A, B)** Summary of the functional categories of genes **(A)** and pathways **(B)** significantly enriched in response to MGCD treatment for seven days. Green bars represent the proportion of genes involved in each category, red curve indicates statistical significance. **(C)** Venn diagram showing overlap of differentially expressed genes from D7M versus D7C (orange circle) and D3M versus D3C (blue circle). **(D, E)** Summary of the functional categories of genes **(D)** and pathways **(E)** enriched both in D7M versus D7C and D3M versus D3C groups. Green bars represent the proportion of genes involved in each category. Red curve indicates statistical significance. GO, gene ontology.

To elucidate potential molecular networks regulated by HDACs, we performed gene expression profiling of the differentiating cells treated with MGCD on day 3 and day 7 (D3M and D7M), as well as the control cells at the same time points of neural differentiation (D3C and D7C). A total of 1,632 genes were differentially expressed between the MGCD-treated group and the control group on day 3 of differentiation (D3M versus D3C), whereas at the end of this stage, 2,475 genes were differentially expressed (D7M versus D7C) (Figure 4A-C). Among them, we found that 399 genes were differentially expressed consistently from day 3 to day 7 after MGCD treatment (Figure 4C). Gene ontology (GO) analysis of the overlapped genes showed that the genes associated with organismal development, cell differentiation and nervous system development were significantly affected by MGCD treatment during differentiation (Figure 4D). Furthermore, pathway enrichment analysis of the overlapped genes revealed that multiple signaling pathways relevant to neural differentiation, such as phosphatidylinositol 3-kinase (PI3K)-Akt, mitogen-activated protein kinase (MAPK) and Notch signaling pathway, were significantly activated by MGCD (Figure 4E).

**Figure 5:**
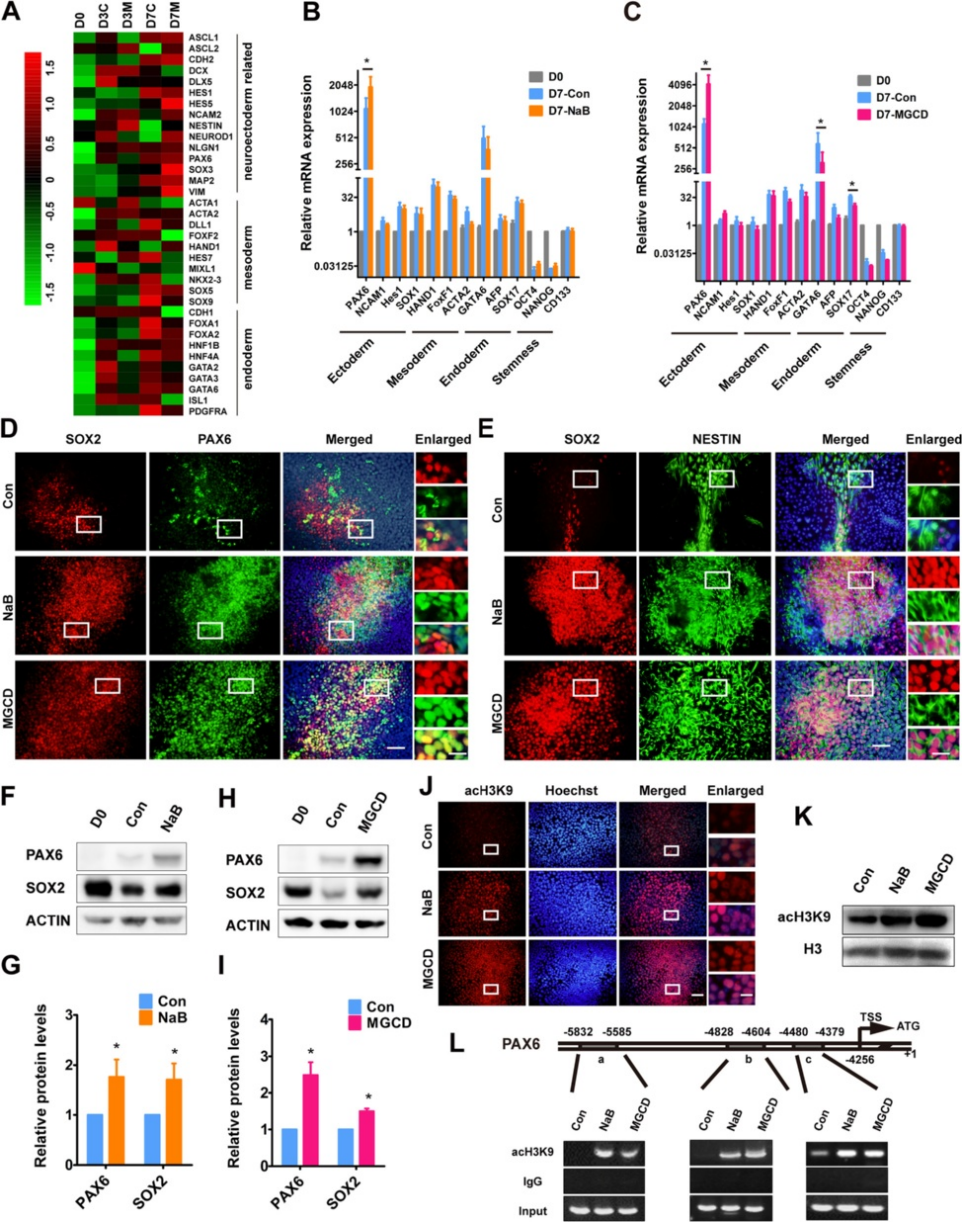
**HDACi greatly promote neuroectodermal specification. (A)** Heat map of normalized expression levels of the typical neuroectoderm, mesoderm and endoderm genes which were differentially expressed after MGCD treatment for three days or seven days. Low expression levels are indicated by green colors, and high expression levels are indicated by red colors. **(B** **,** **C)** On day 7 of differentiation, real-time PCR was performed to determine the mRNA levels of typical genes for three germ layers after NaB or MGCD treatment for seven days. **(D,** **E)** On day 7, neuroectodermal cells were visualized by immunostaining with antibodies against SOX2 (red) and PAX6 (green) **(D)** or SOX2 (red) and NESTIN (green) **(E)**. Scale bars, 50 μm. Higher magnification views are shown in the inset frames. Scale bars, 150 μm. **(F-I)** Protein levels of PAX6 and SOX2 were assessed by immunoblotting. The density of the bands was analyzed by the Image J software and expressed as fold of control. **(J,** **K)** Acetylation levels of histone H3K9 were detected by immunostaining **(J)** and immunoblotting **(K)**, respectively. Higher magnification views are shown in the inset frames. Scale bars, 150 μm. **(L)** H9 cells were cultured in the ESC medium with the addition of NaB or MGCD for seven days and immunoprecipitated with anti-acH3K9 antibody. ChIP DNA levels of PAX6 were analyzed by semi-quantitative PCR. Scale bars, 50 μm. The error bars indicate SEM, *P <0.05; n >/=3. ChIP, chromatin immunoprecipitation; HDACi, HDAC inhibitors; SEM, standard error of the mean.

From the results of both genome-wide expression analysis and real-time PCR, we found that compared with undifferentiated H9, the transcripts of stemness markers were decreased during differentiation, while the transcripts of the markers of the three germ layers were increased, suggesting that the cells with different fates were specified from H9 after RA induction for seven days (Figure 5A-C).

We assumed that suppression of histone deacetylation might assist RA to induce H9 cells to differentiate towards neuroectodermal cells during the first seven days. Interestingly, NaB and MGCD treatment increased the transcripts of the majority of neuroectodermal markers (for example, *PAX6*), while it decreased the transcripts of mesodermal and endodermal markers (for example, *GATA6*) (Figure 5A-C). Noticeably, the transcript of *PAX6* was increased dramatically after RA treatment and reached a higher level with the addition of NaB or MGCD (Figure 5B and C). Moreover, we analyzed the cells by immunostaining and flow cytometry with antibodies against PAX6, SOX2 and NESTIN on day 7 of differentiation. We found that the number of dual positive PAX6-SOX2 cells or SOX2-NESTIN cells was significantly increased after NaB and MGCD treatment at the end of the RA induction stage (Figure 5D and E; Additional file 3: Figure S3). Similarly, the protein levels of PAX6 and SOX2 in the HDACi-treated cells were significantly higher than in the control cells (Figure 5F-I), confirming that HDACi promote NPC differentiation. These results indicate that HDACi might promote neuroectodermal specification at the early stage of differentiation.

We then asked how NaB and MGCD enhanced neuroectodermal specification. It is known that HDACi can induce hyperacetylation of histones by inhibiting histone deacetylation. We found that the acetylation levels of histone H3 at lysine 9 (acH3K9) in the NaB- and MGCD-treated cells were higher than that of the control cells on day 7 of differentiation (Figure 5J and K). The increased expression of neuroectodermal genes might be directly related to acH3K9. We performed chromatin immunoprecipitation (ChIP) and found that the binding of acetylated histone H3 with the promoter of *PAX6* was significantly enhanced in the NaB- and MGCD-treated cells, which may contribute to the increased expression of *PAX6* (Figure 5L).

Taken together, these results suggest that inhibiting histone deacetylation by HDACi could promote the expression of *PAX6* and other neuroectodermal genes and give rise to more cells with a neural cell lineage-fate during RA treatment, thus generating more NPCs.

### HDACi promote the differentiation of NPCs from hiPSCs

**Figure 6:**
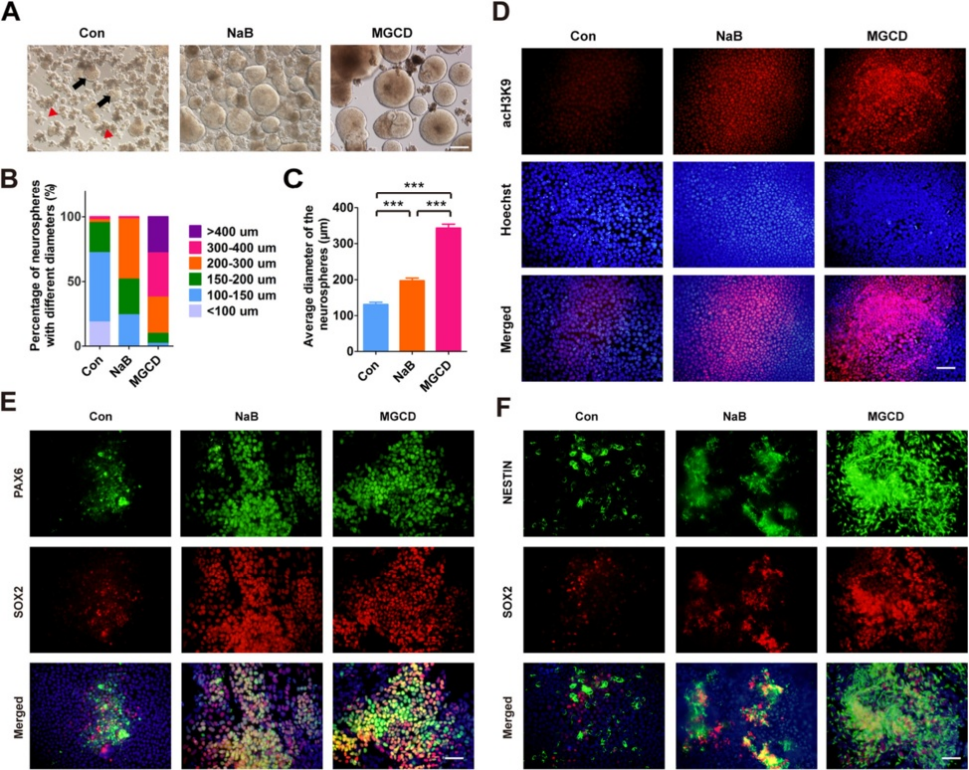
**HDACi enhance the NPC generation of hiPSCs. (A-C)** Neurospheres were formed from the N-iPSC-1 cell line after HDACi treatment and the diameters of the neurospheres were measured and quantified on day 18. The error bars indicate SEM, ***P <0.001; n >/=50. Black arrows represent typical neurospheres and red arrowheads represent cell debris. **(D)** Acetylation levels of histone H3K9 in N-iPSC-1 cells were detected by immunostaining on day 7 of differentiation. **(E,** **F)** Neuroectodermal cells were visualized by immunostaining with antibodies against PAX6 (green) and SOX2 (red) **(E)** or NESTIN (green) and SOX2 (red) **(F)** on day 7 of differentiation. Scale bars, 50 μm. HDACi, HDAC inhibitors; hiPSCs, human induced pluripotent stem cells; SEM, standard error of the mean.

To investigate whether HDACs can regulate the differentiation of NPCs from other hPSCs, we employed an hiPSC line (named N-iPSC-1) which was kindly provided by Dr. Ying Jin’s lab [26]. We found that NaB and MGCD also enhanced the formation of neurospheres of N-iPSC-1 (Figure 6A). The neurospheres derived from HDACi treatment were significantly larger than controls (Figure 6B and C), and the histone acetylation levels of the HDACi-treated cells were higher than that of the control cells on day 7 of differentiation (Figure 6D). Meanwhile, there were apparently more PAX6^+^, SOX2^+^ and NESTIN^+^ cells in the HDACi- treated cells (Figure 6E and F), indicating an increase in conversion of neuroectodermal cells after HDAC was inhibited. These results are consistent with those of H9, confirming that inhibiting HDACs can promote differentiation of NPCs not only from hESCs but also from hiPSCs in a similar manner.

### Knockdown of HDAC3 or SMRT enhances NPC generation

**Figure 7:**
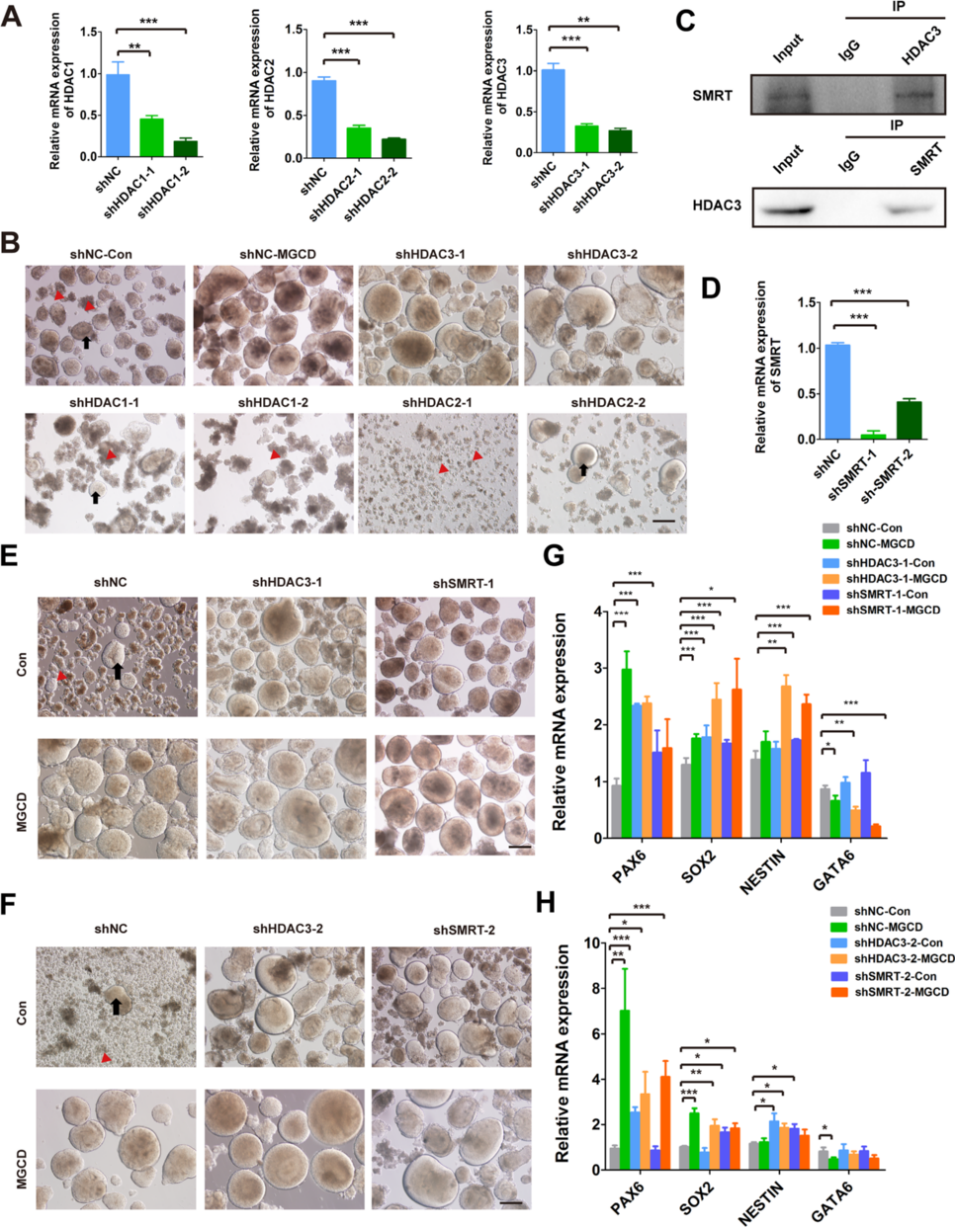
**Knockdown of HDAC3 or SMRT enhances NPC generation. (A)** Knockdown efficiency of shHDAC1 or shHDAC2, shHDAC3 was assessed by real-time PCR. The error bars indicate SEM. **P <0.01; ***P <0.001. **(B)** Neurosphere formation of H9-shHDAC1 and H9-shHDAC2 cells as well as H9-shHDAC3 cells were analyzed on day 18. Scale bar, 200 μm. Black arrows represent typical neurospheres, and red arrowheads represent cell debris. **(C)** Co-IP of endogenous HDAC3 and SMRT from H9 cell lysate was performed with HDAC3 or SMRT antibodies. **(D)** Knockdown efficiency of SMRT was assessed by real-time PCR. **(E, F)** On day 18 of differentiation, neurospheres were formed in shNC, shHDAC3 and shSMRT H9 cells with or without MGCD treatment for seven days. Black arrows represent typical neurospheres, and red arrowheads represent cell debris. **(G, H)** On day 7 of differentiation, real-time PCR was performed to determine the mRNA levels of neuroectodermal genes (PAX6, SOX2 and NESTIN) and endoderm gene (GATA6) in shHDAC3 and shSMRT H9 cells with or without MGCD treatment for seven days. Scale bars, 200 μm. The error bars indicate SEM, *P <0.05; **P <0.01; ***P <0.001; n >/=3. Co-IP, co-immunoprecipitation; HDAC, histone deacetylase; NPC, neural progenitor cell; SEM, standard error of the mean.

We then attempted to identify which HDAC critically functioned in NPC generation. We knocked down HDAC1, HDAC2 or HDAC3 individually by several short hairpin RNA (shRNA) sequences and used one non-target shRNA sequence as a negative control (shNC). We found that the mRNA and protein levels of the *HDACs* were significantly decreased in the transfected cells (Figure 7A; Additional file 4: Figure S4A-S4D). Interestingly, knockdown of HDAC3 but not HDAC1 or HDAC2 could enhance neurosphere formation as MGCD treatment (Figure 7B). Although HDAC1 and HDAC2 were also the targets of MGCD, there was apparently more cell debris and fewer neurospheres derived from H9-shHDAC1 cells or even worse in H9-shHDAC2 cells. We observed that the transcript levels of PAX6 and SOX2 were down-regulated whereas the GATA6 level was up-regulated after HDAC1 was suppressed, which is just the opposite of the results of MGCD treatment (see Additional file 4: Figure S4E). These results suggest that HDAC3 but not HDAC1 or HDAC2 is the critical regulator that negatively regulates NPC generation.

It has been demonstrated that HDACs execute their functions by forming multiprotein complexes with their cofactors. Generally, HDAC1 and HDAC2 are recruited to the corepressors REST/CoREST while HDAC3 is recruited to NCoR/SMRT. The complexes then bind to sequence-specific DNA, which can condense the chromatin structures and repress gene expression [27]. We demonstrated that HDAC3 interacted with SMRT in H9 by co-immunoprecipitation (Co-IP) assay (Figure 7C). We speculated that HDAC3 might form this complex with SMRT in the NPC commitment. To confirm this assumption, we knocked down SMRT in H9 cells. The expression of SMRT was down-regulated in undifferentiated H9 and it remained lower in H9-shSMRT cells after seven days of differentiation, as was the expression of HDAC3 in H9-shHDAC3 cells (Figure 7D; Additional file 4: Figure S4F). Excitingly, the number and size of neurospheres generated from H9-shSMRT cell lines were comparable to those from H9-shHDAC3 cell lines (The upper panels of Figure 7E and F). Also, the expression levels of *PAX6*, *SOX2* and *NESTIN* were significantly higher in H9-shHDAC3 (H9-shHDAC3-Con) and H9-shSMRT (H9-shSMRT-Con) cells than in H9-shNC (H9-shNC-Con) cells at the end of the first stage, which is consistent with the results of MGCD treatment (H9-shNC-MGCD) (Figure 7G and H; Additional file 4: Figure S4G). Moreover, upon MGCD treatment, H9-shHDAC3 cells and H9-shSMRT cells showed a further increase in neurosphere formation during differentiation (the lower panels of Figure 7E and F) and an up-regulation in transcripts of *PAX6*, *SOX2* and *NESTIN,* but a down-regulation in transcript of *GATA6* (Figure 7G and H), implying that the inhibitor treatment and knockdown of the repressors might have a synergistic effect on NPC generation.

We then asked whether knockdown of *HDAC3* or *SMRT* may alter the identity of H9. We found that the profiles of SOX2 and OCT4 immunostaining in H9-shHDAC3 or H9-shSMRT cells were similar to those in H9-shNC (Additional file 4: Figure S4H). These results suggest that suppression of HDAC3 or SMRT can enhance neuroectodermal specification without compromising the identity of hPSCs.

**Figure 8:**
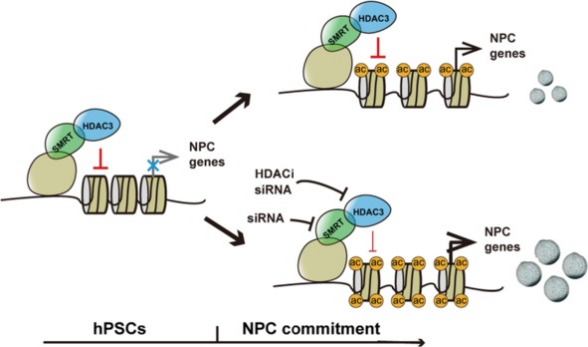
**Proposed model of enhanced NPC generation by inhibiting the HDAC3/SMRT co-repressor complex.** NPC, neural progenitor cell.

Overall, our study reveals that NPC specification from hPSCs can be repressed by the complex of HDAC3 and its co-repressor SMRT. During the process of neural differentiation, the repression of neuroectodermal genes is attenuated, which is accompanied by an increase in acetylation of target genes (Figure 8). Furthermore, HDACi treatment or knockdown of the repressors may further reduce this repression and then promote the hPSC differentiation into NPCs (Figure 8).

## Discussion

HDACs demonstrate very pleiotropic ability in stem cell differentiation, including acting on NSC, hematopoietic stem cell and cardiac stem cell differentiation [28-30]. Previous investigations reported that inhibiting HDACs by HDACi could promote NSCs and NPCs to differentiate into neuronal or glial cells [17,22,31,32]. However, the commitment of NPCs from hPSCs and further differentiation of NPCs into neuronal or glial cells are two successive but different processes regulated by different mechanisms [33-35].

Here we report that HDACs participate in the earlier process of neural differentiation. It has been reported that PAX6 and SOX2 control NPC identity [36-38], and PAX6 is an important transcriptional determinant of human neuroectodermal cell fate; knockdown of PAX6 blocks neuroectodermal specification from hPSCs [39]. Thus, the up-regulation of PAX6 and SOX2 might account for the conversion of more cells with a neuroectodermal fate. In addition, the down-regulated transcripts of mesodermal and endodermal markers in HDACi-treatment cells during the RA induction stage represent a reduction of specification of mesoderm and endoderm cells, which may lead to increased conversion of hPSCs towards ectoderm cells. Together, the change of cell population at the beginning of neurosphere formation might be one of the reasons that lead to increased NPC generation. However, there also exists another possibility that all PSCs are directed to differentiate to NPC under the induction of RA. However, neural differentiation is a dynamic process, and not all cells can differentiate in a synchronous manner even under the same neural differentiation condition [40,41]. Thus, only parts of PSCs can finally commit to NPCs, while many cells die at the beginning of neurosphere formation. Upon HDAC suppression, the expression levels of NPC markers in all cells are increased, and, therefore, cells are more prone to commit to NPCs under that differentiation condition.

We observe that knockdown of HDAC3 in H9 cells shows similar results with those of HDACi treatment, whereas suppression of HDAC1 or HDAC2 fails to enhance the NPC specification. Indeed, there are apparently more dead cells and fewer neurospheres derived from H9-shHDAC1 and H9-shHDAC2 than from H9-shNC during differentiation, suggesting that HDAC1 or HDAC2 is more likely to function in cell survival or other key cellular events. It has been reported that HDAC1 deletion causes embryonic lethality before embryonic day (E)10.5 in mice [42] and half of HDAC2-null mice succumb by postnatal day 25 [43]. Moreover, conditional deletion of both HDAC1 and HDAC2 in NSCs in mice results in severe brain abnormalities and lethality at postnatal day 7 [44]. Previous studies also showed that deleted HDAC3 could lead to deficiency in neural crest development in mice [45], and activation of HDAC3 could enhance endothelial cell differentiation from ESCs [46]. It seems that HDACs have essential, but redundant, functions during neural system development. Robustly genetic loss of HDACs may block the process of neural differentiation in mouse brain and lead to developmental defects, whereas inhibition of HDACs to some extent could enhance the NPC commitment.

It is known that neuronal genes can be repressed by the complexes of HDACs and their co-repressors in mouse ESCs, NPCs and differentiated non-neuronal cells [47-49]. Here we report that individually knocking down any factor of the HDAC3/SMRT complex can elevate the expressions of neuroectodermal genes, indicating that HDAC3 might negatively regulate the NPC commitment through the repressor complex with SMRT. SMRT has been shown to inhibit the Notch signaling pathway by repressing the transcription of *HES1* [50,51]. The whole-genome expression analysis in our study reveals that the Notch signaling pathway is influenced by MGCD treatment. It is likely that the HDAC3/SMRT complex regulates NPC generation by modifying Notch signaling pathway. Future studies are needed to further determine the downstream target genes of HDAC3/SMRT and how this complex regulates NPC specification.

Furthermore, our findings may also facilitate the establishment of an optimized strategy of neural differentiation from hPSCs for future studies. We are able to enhance the generation of NPCs from hPSCs by 10- to 15-fold through the application of HDACi. Although knockdown of HDAC3 or SMRT can promote NPC generation, pharmacological inhibition of HDACs is more practical for future clinical application. Importantly, HDACi treatment other than knockdown of HDACs can keep genetic fidelity of the cells during differentiation. The similar outcomes from the hiPSC line, N-iPSC-1, show that the initial epigenetic landscapes between H9 and N-iPSC-1, at least the deacetylation landscapes for neural differentiation, are very similar. It is believed that NPCs from patient-specific iPSCs are better suited for the transplantation treatment of multiple neurodegenerative diseases. Our unpublished data show that the NPC generation of Alzheimer’s disease (AD) patient-specific iPSCs can also be enhanced by HDACi treatment. This might imply a broader application of our findings in iPSCs derived from patients with specific neurodegenerative diseases. Therefore, our study may provide useful information for further investigation of the molecular mechanisms underlying the induction of specific NPCs from hPSCs and may facilitate their application in NPC-transplantation therapies.

## Conclusions

In summary, our findings demonstrate that HDACs, in particular HDAC3, negatively regulate the differentiation of NPCs from hPSCs. HDAC3 might orchestrate SMRT to form a repressor complex, inhibition of which promotes NPC specification. Taken together, our data provide a novel regulatory epigenetic mechanism in governing the differentiation of hPSCs towards NPCs.

## Methods

### Cell culture of hPSCs

Human ESCs H9 were purchased from WiCell (Madison, WI, US) and human iPSCs (named N-iPSC-1) [26] were provided by Dr. Ying Jin from the Institute of Health Sciences, Shanghai Institutes for Biological Sciences. Both cell lines were cultured in the mTeSR1 medium (STEMCELL Technologies, Vancouver, BC, Canada) on matrigel (BD, Franklin Lakes, NJ, USA) coated dishes. H9 and N-iPSC-1 lines with passage number P35 to P55 were used in this study*.*

### NPC generation and differentiation

Neural differentiation was induced by RA treatment. First, a total 1 × 10^6^ undifferentiated hPSCs split into small clones by dispase (1 mg/ml, Millipore, Billerica, MA, USA) were cultured in mTeSR1 medium with 10 μM all-*trans*-retinoic acid (Sigma, St.Louis, MO, USA) on a matrigel-coated 6-cm dish. The cell numbers of the small clones varied from about 340 to 870 cells (520 cells average) as we show in Additional file 1: Figure S1A and Additional file 4: Figure S4H. After seven days of RA treatment, the cells were digested by dispase and gently pipetted into small clumps, the sizes of which were comparable to the ESC clones when we initiated neural differentiation. The cell clumps were aggregated in knock-out serum replacement (KSR) medium for four days, followed by culture in neurosphere medium for seven more days to form neurospheres. After that, neurospheres were digested into single cells by accutase (STEMCELL Technologies), which can either be maintained as NPCs in NPC medium or further be induced to neurons or glia.

In some cases, HDACi was added to the mTeSR1 with RA for a different number of days at the first stage. NaB and VPA were purchased from Sigma; MGCD, TubA and PCI were from Selleck Chemicals (Houston, TX, USA).

NPCs were spontaneously differentiated into neurons or glia in NPC medium without bFGF. MAP2^+^ neurons and GFAP^+^ glia could be detected after differentiated for more than 35 days.

For neuronal differentiation with high efficiency, NPCs were plated on poly-L-ornithine (PLO, 15 μg/ml, Sigma)/Laminin (1 μg/ml, Sigma)-coated dishes at a density of 1 × 10^4^ cells/cm^2^ in the neuronal differentiation medium for neuron generation.

### Medium for differentiation

KSR medium: (D)MEM/F12 medium with 20% KSR, 1% GlutaMax-1, 1% non-essential amino acid (NEAA), 50 μM β-mercaptoethanol (ME) and 1% penicillin/streptomycin (P/S).

Neurosphere medium: (D)MEM/F12 medium containing 1% N2 supplement, 1% GlutaMax-1, 1% NEAA, 50 μM β-ME, 1% P/S, 8 μg/ml heparin, 20 ng/ml bFGF and 20 ng/ml epidermal growth factor (EGF).

NPC medium: (D)MEM/F12 and neurobasal medium (1:1) containing 0.5% N2, 1% B27, 1% GlutaMax-1, 1% NEAA, 50 μM β-ME, 1% P/S and 10 ng/ml bFGF.

Neuronal differentiation medium: neurobasal medium containing 1% N2, 2% B27, 1% GlutaMax-1, 1% NEAA, 50 μM β-ME, 1% P/S, 20 ng/ml never growth factor (NGF), 20 ng/ml brain-derived neurotrophic factor (BDNF) and 20 ng/ml glial-eerived neurotrophic factor (GDNF).

The medium and supplements were from Invitrogen (Carlsbad, NY, USA). All growth factors and cytokines were from R&D (Minneapolis, MN, USA).

### Clones and lentivirus packaging

Lentiviral vectors targeting human *HDAC1*, *HDAC2* and *HDAC3* were purchased from 3D-HTS company (Shanghai, China) and validated in 293 T cells. The pLKO.1 lentiviral vector was employed to knockdown human *SMRT*. Briefly, siRNA oligos targeting *SMRT* were annealed and inserted into the A*ge*I and E*co*RI sites of the pLKO.1 vector. The targeted sequences of SMRT were: 5′-GCT TCA CAA CAC AGG CAT GAA-3′ and 5′-GCA GCG CAT CAA GTT CAT CAA-3′. For packaging, 293FT cells were co-transfected with the lentiviral vector and two helper plasmids pMD.G and psPAX2 by calcium phosphate transfection according to a previously described method [52,53]. The medium was then replaced after overnight. The supernatant was harvested 48 and 72 hours, respectively, after transfection and concentrated by precipitation with PEG-8000 (Sigma). The yielded lentivirus particles were then dissolved in PBS, aliquoted and frozen at −80°C until use. Generally, every 10 ml of supernatant was finally concentrated into 50 μl as one aliquot.

### Stably-transfected cell line screening

H9 cells were digested into single cells by accutase and plated in 6-well-plates at a density of 1 × 10^6^ cells/well in mTeSR1 medium. After adherence, one aliquot of lentivirus particles was applied to the cells for 24 hours. The medium was then changed every day and Y-27632 (STEMCELL Technologies) was added to the medium for the first two days. After 72 hours, 0.5 μg/ml of puromycin (Millipore) was used to select the transfected cells.

### Gene expression

Total RNA was isolated from cultured cells using TRIzol (Invitrogen) and was reverse-transcripted into cDNA using a ReverTra Ace qPCR RT Kit (Toyobo, Osaka, Japan). Real-time PCR was then performed using a SYBR premix Ex TaqTM II kit (Takara, Shiga, Japan) and carried out on a real-time PCR System (ABI 7500). All primers were verified by melting curve analyses containing a single melt curve peak. Primer information is listed in Additional file 5: Table S1. The relative expression level of each gene was analyzed using the 2^-ΔΔCt^ algorithm normalizing to GAPDH and relative to the control samples.

### Flow cytometry analysis

For flow cytometry analysis of NPCs, cells of the neurospheres derived from H9 were fixed by 4% paraformaldehyde (PFA) for 10 minutes, and then blocked in PBS with 0.5% BSA and 0.1% Triton X-100 for 30 minutes. After that, cells were stained by Alexa fluor-647 mouse anti-NESTIN (1:500, BD), Alexa fluor-488 mouse anti-SOX2 (1:100, BD) or PerCP-Cy™ 5.5 mouse anti-human PAX6 (1:50, BD) for one hour. Finally, NPCs were washed twice with PBS and then suspended in 200 μl of PBS for flow cytometry analysis (BD FACSAria).

The proliferation ability was measured by flow cytometry analysis. Cells from H9 on day 3 of differentiation were incorporated with BrdU (1:100, Sigma) in a cell incubator for 30 minutes and digested into single cells for fixation in 4% PFA for 10 minutes, and then treated with 2 M HCL for 30 minutes at 37°C, followed by Alexa-fluor-647 mouse-anti-BrdU (1:100, Sigma) staining as described above.

The number of apoptotic cells were measured using an Annexin V apoptosis detection kit by flow cytometry according to the manufacturer’s instructions (eBioscience, San Diego, CA, USA).

### Immunoblotting

Total proteins were extracted from cultured cells using radioimmunoprecipitation assay (RIPA) lysis buffer (Beyotime, Jiangsu, China) and 30 μg proteins were then loaded and separated by SDS-PAGE gels, followed by transfer onto polyvinylidene fluoride (PVDF) membranes, and incubation of the membranes with 5% nonfat milk for one hour at room temperature to block nonspecific sites. After that, the membranes were incubated overnight at 4°C with the following primary antibodies: H3 (1:2,000; Cell Signaling, Danvers, MA, USA), acH3K9 (1:1,000; Cell Signaling), PAX6 (1:1,000; Millipore), SOX2 (1:1,000; Cell Signaling), HDAC1 (1:1,000; Cell Signaling), HDAC3 (1:1,000; Cell Signaling), SMRT (1:100, Sigma) and β-actin (1:30,000; Sigma). The membranes were then washed with PBS and incubated with horseradish peroxidase (HRP) conjugated secondary antibodies (1:2,000; Cell Signaling) at room temperature for two hours. The signals were finally detected using a SuperSignal Detection Kit (Pierce, Rockford, IL, USA) according to the manufacturer’s instructions.

### Immunostaining

To perform immunofluorescent staining, cells from hPSCs during differentiation plated on the glass coverslips were fixed in 4% PFA for 15 minutes at room temperature. After washing, the fixed cells were incubated with blocking buffer (PBS containing 3% goat serum and 0.3% Triton X-100) for 30 minutes at room temperature, followed by an overnight incubation at 4°C with the following primary antibodies: OCT4 (1:400, Cell Signaling), PAX6 (1:400; Millipore), SOX2 (1:400; Cell Signaling), NESTIN (1:800; Millipore), MAP2 (1:1 000; Sigma), NeuN (1:500; Millipore) and Ac-H3K9 (1:400; Cell Signaling). After repeated washes, the cells were incubated with secondary antibodies conjugated with Alexa fluor-488/555 (1:400; Invitrogen) at room temperature for two hours. The nuclei were finally stained with Hoechst 33342 (Beyotime) before observation under microscopy (Olympus). For quantitative analysis, the percentage of NPCs or neurons was calculated by the number of NESTIN^+^ or NeuN^+^ cells versus the number of Hoechst^+^ cells. For each treated group, more than three random images were taken and the number of cells were counted, and the results were determined from more than four independent experiments.

### Chromatin immunoprecipitation assay

A ChIP assay was carried out using a Pierce Agarose ChIP Kit according to the manufacturer’s instructions (Thermo Scientific, Rockford, IL, USA). Briefly, 2 × 10^6^ cells were cross-linked in 1% (w/v) formaldehyde for 10 minutes at room temperature and inactivated by the addition of glycine. Cells were then washed and harvested into tubes by scraping before centrifugation. After that, cell pellets were lysed and the nuclei were digested with Micrococcal Nuclease. The sheared chromatin extracts were immunoprecipitated with anti-acH3K9 (1:50; Cell Signaling) antibody at 4°C overnight, followed by incubation with A/G plus agarose resin. The binding DNA was then recovered after incubation at 65°C and purified as templates for semi-quantitative PCR. The primers specific to the *PAX6* promoter are listed in Additional file 5: Table S1.

### Co-immunoprecipitation assay

For validation of the interaction between HDAC3 and SMRT, H9 cells were digested by IP lysis buffer (Beyotime) with 1% PMSF and the cell extracts were pre-cleared by incubating with 20 μl protein A/G agarose (Santa Cruz, Dallas, TX, USA) at 4°C for 30 minutes and the supernatant was transferred to new 1.5 ml tubes. Then the pre-cleared cell extracts were incubated with 1 μg HDAC3 antibody (Cell Signaling), 2 μg SMRT antibody (Santa Cruz) or 1 μg rabbit IgG (Cell signaling), respectively, at 4°C for two hours. Immunocomplexes were then crosslinked with 30 μl protein A/G agarose at 4°C overnight. The tubes were spun and the bound proteins (immunoprecipitates) were washed five times with IP lysis buffer containing 1% PMSF at 4°C, and eluted from beads by incubation with 1 × SDS protein loading buffer. Input and IP protein samples were analyzed by immunoblotting.

### Microarray analysis

A total of five mRNA samples were prepared for whole-genome microarray (Affymetrix U133 plus 2.0 gene chips) analysis at the Shanghai Biotechnology Corporation. Among these samples, one was undifferentiated H9 cells (D0), two were the control H9 cells on day 3 and day 7 of neural differentiation (D3C and D7C), and another two were MGCD-treated H9 cells at the two time points of neural differentiation (D3M and D7M). Global differentially expressed genes between control cells and MGCD-treated cells from each day of differentiation were analyzed by GO analysis and pathway enrichment analysis as previously described [54]. We have deposited our raw microarray data and the results of differentially expressed genes, GO analysis as well as pathway enrichment analysis in the Gene Expression Omnibus (GEO) of the National Center for Biotechnology Information [55] with GEO accession number GSE61050.

### Statistical analysis

All values are presented as the mean ± SEM values from more than three independent experiments. Statistical significance was determined using student’s *t*-test by GraphPad Prism. The results were considered significant when the *P*-value was less than 0.05.

## Additional files

Additional file 1: Figure S1 HDACs participate in the process of neural differentiation. **(A)** Immunostaining of undifferentiated H9 cells with antibody against OCT4 (green), SOX2 (red), NESTIN (red) and PAX6 (red). Scale bar, 50 μm. **(B)** The mRNA expressions of class I and class II HDACs during neural differentiation were determined by real-time PCR. The error bars indicate SEM. Additional file 2: Figure S2 Concentrations of NaB and VPA and time-dependent manner of MGCD. **(A)** The morphology of H9 cells was observed after treatment with various concentrations of NaB or VPA. **(B)** Neurospheres were formed from H9 cells that were induced for neural differentiation with the addition of MGCD for two, three, four and five days, respectively. Scale bar, 200 μm. Additional file 3: Figure S3 Flow cytometry analysis of the cells on day 7 of differentiation. **(A, B)** PAX6 and SOX2 dual positive cells were analyzed and quantified by FC. The error bars indicate SEM, ****P* <0.001; n = 3. **(C, D)** NESTIN and SOX2 dual positive cells were analyzed and quantified by flow cytometry. The error bars indicate SEM, ****P* <0.001; n = 4. Additional file 4: Figure S4 Neurosphere formation and stemness after HDAC or SMRT suppression. **(A-D)** Knockdown efficiency of HDAC1 or HDAC3 was determined by western blot. The density of the bands was analyzed using Image J software and expressed as fold of control. **(E)** The transcripts of PAX6, SOX2, NESTIN and GATA6 in H9-shHDAC1 cells on day 7 of differentiation. **(F)** Expression of HDAC3 or SMRT in H9-shHDAC3 or H9-shSMRT on day 7 of differentiation. **(G)** Protein levels of PAX6 and SOX2 were assessed by western blot in shHDAC3 cells with or without MGCD treatment on day 7 of differentiation. **(H)** Stemness of undifferentiated H9-shHDAC3 or H9-shSMRT cells was detected by immunostaining with anti-OCT4 (green) and anti-SOX2 (red) antibodies. Scale bar, 50 μm. The error bars indicate SEM. **P* <0.05; ***P* <0.01; ****P* <0.001; n>/=3. Additional file 5: Table S1 A list of primers used for real-time PCR and ChIP analysis.

## References

[1] Thomson JA (1998). Embryonic stem cell lines derived from human blastocysts. Science.

[2] Takahashi K, Tanabe K, Ohnuki M, Narita M, Ichisaka T, Tomoda K, Yamanaka S (2007). Induction of pluripotent stem cells from adult human fibroblasts by defined factors. Cell.

[3] Yu JY, Vodyanik MA, Smuga-Otto K, Antosiewicz-Bourget J, Frane JL, Tian S, Nie J, Jonsdottir GA, Ruotti V, Stewart R, Slukvin II, Thomson JA (2007). Induced pluripotent stem cell lines derived from human somatic cells. Science.

[4] Roy NS, Cleren C, Singh SK, Yang L, Beal MF, Goldman SA (2006). Functional engraftment of human ES cell-derived dopaminergic neurons enriched by coculture with telomerase-immortalized midbrain astrocytes. Nat Med.

[5] Dhara SK, Stice SL (2008). Neural differentiation of human embryonic stem cells. J Cell Biochem.

[6] Yu DX, Marchetto MC, Gage FH (2013). Therapeutic translation of iPSCs for treating neurological disease. Cell Stem Cell.

[7] Maden M (2007). Retinoic acid in the development, regeneration and maintenance of the nervous system. Nat Rev Neurosci.

[8] Erceg S, Lainez S, Ronaghi M, Stojkovic P, Perez-Arago MA, Moreno-Manzano V, Moreno-Palanques R, Planells-Cases R, Stojkovic M: **Differentiation of human embryonic stem cells to regional specific neural precursors in chemically defined medium conditions.** *PLoS One* 2008, **3:**e2122. 10.1371/journal.pone.0002122 PMC2346555 18461168

[9] Okada Y, Shimazaki T, Sobue G, Okano H (2004). Retinoic-acid-concentration-dependent acquisition of neural cell identity during in vitro differentiation of mouse embryonic stem cells. Dev Biol.

[10] Patthey C, Edlund T, Gunhaga L (2009). Wnt-regulated temporal control of BMP exposure directs the choice between neural plate border and epidermal fate. Development.

[11] Monsoro-Burq AH, Fletcher RB, Harland RM (2003). Neural crest induction by paraxial mesoderm in Xenopus embryos requires FGF signals. Development.

[12] Garcia-Castro MI, Marcelle C, Bronner-Fraser M (2002). Ectodermal Wnt function as a neural crest inducer. Science.

[13] Chambers SM, Fasano CA, Papapetrou EP, Tomishima M, Sadelain M, Studer L (2009). Highly efficient neural conversion of human ES and iPS cells by dual inhibition of SMAD signaling. Nat Biotechnol.

[14] Ying QL, Stavridis M, Griffiths D, Li M, Smith A (2003). Conversion of embryonic stem cells into neuroectodermal precursors in adherent monoculture. Nat Biotechnol.

[15] Sikorska M, Sandhu JK, Deb-Rinker P, Jezierski A, LeBlanc J, Charlebois C, Ribecco-Lutkiewicz M, Bani-Yaghoub M, Walker PR (2008). Epigenetic modifications of SOX2 enhancers, SRR1 and SRR2, correlate with in vitro neural differentiation. J Neurosci Res.

[16] Hermanson O, Jepsen K, Rosenfeld MG (2002). N-CoR controls differentiation of neural stem cells into astrocytes. Nature.

[17] Hsieh J, Nakashima K, Kuwabara T, Mejia E, Gage FH (2004). Histone deacetylase inhibition-mediated neuronal differentiation of multipotent adult neural progenitor cells. Proc Natl Acad Sci U S A.

[18] Humphrey GW, Wang YH, Hirai T, Padmanabhan R, Panchision DM, Newell LF, McKay RD, Howard BH (2008). Complementary roles for histone deacetylases 1, 2, and 3 in differentiation of pluripotent stem cells. Differentiation.

[19] Rossler R, Boddeke E, Copray S (2010). Differentiation of non-mesencephalic neural stem cells towards dopaminergic neurons. Neuroscience.

[20] Tapias A, Zhou ZW, Shi Y, Chong Z, Wang P, Groth M, Platzer M, Huttner W, Herceg Z, Yang YG, Wang ZQ (2014). Trrap-dependent histone acetylation specifically regulates cell-cycle gene transcription to control neural progenitor fate decisions. Cell Stem Cell.

[21] Lane MA, Bailey SJ (2005). Role of retinoid signalling in the adult brain. Prog Neurobiol.

[22] Yu IT, Park JY, Kim SH, Lee JS, Kim YS, Son H (2009). Valproic acid promotes neuronal differentiation by induction of proneural factors in association with H4 acetylation. Neuropharmacology.

[23] Lyssiotis CA, Walker J, Wu C, Kondo T, Schultz PG, Wu X (2007). Inhibition of histone deacetylase activity induces developmental plasticity in oligodendrocyte precursor cells. Proc Natl Acad Sci U S A.

[24] Balasubramanian S, Ramos J, Luo W, Sirisawad M, Verner E, Buggy JJ (2008). A novel histone deacetylase 8 (HDAC8)-specific inhibitor PCI-34051 induces apoptosis in T-cell lymphomas. Leukemia.

[25] Fournel M, Bonfils C, Hou Y, Yan PT, Trachy-Bourget MC, Kalita A, Liu J, Lu AH, Zhou NZ, Robert MF, Gillespie J, Wang JJ, Ste-Croix H, Rahil J, Lefebvre S, Moradei O, Delorme D, Macleod AR, Besterman JM, Li Z (2008). MGCD0103, a novel isotype-selective histone deacetylase inhibitor, has broad spectrum antitumor activity in vitro and in vivo. Mol Cancer Ther.

[26] Ma Y, Li CL, Gu JJ, Tang F, Li C, Li P, Ping P, Yang S, Li Z, Jin Y (2012). Aberrant gene expression profiles in pluripotent stem cells induced from fibroblasts of a Klinefelter syndrome patient. J Biol Chem.

[27] Yang XJ, Seto E (2008). The Rpd3/Hda1 family of lysine deacetylases: from bacteria and yeast to mice and men. Nat Rev Mol Cell Biol.

[28] Zhang CL, McKinsey TA, Chang SR, Antos CL, Hill JA, Olson EN (2002). Class II histone deacetylases act as signal-responsive repressors of cardiac hypertrophy. Cell.

[29] Chang SR, McKinsey TA, Zhang CL, Richardson JA, Hill JA, Olson EN (2004). Histone deacetylases 5 and 9 govern responsiveness of the heart to a subset of stress signals and play redundant roles in heart development. Mol Cell Biol.

[30] Kretsovali A, Hadjimichael C, Charmpilas N: **Histone deacetylase inhibitors in cell pluripotency, differentiation, and reprogramming.** *Stem Cells Int* 2012, **2012:**184154. 10.1155/2012/184154 PMC3328162 22550500

[31] Siebzehnrubl FA, Buslei R, Eyupoglu IY, Seufert S, Hahnen E, Blumcke I (2007). Histone deacetylase inhibitors increase neuronal differentiation in adult forebrain precursor cells. Exp Brain Res.

[32] Balasubramaniyan V, Boddeke E, Bakels R, Kust B, Kooistra S, Veneman A, Copray S (2006). Effects of histone deacetylation inhibition on neuronal differentiation of embryonic mouse neural stem cells. Neuroscience.

[33] Murry CE, Keller G (2008). Differentiation of embryonic stem cells to clinically relevant populations: lessons from embryonic development. Cell.

[34] Liem KF, Tremml G, Roelink H, Jessell TM (1995). Dorsal differentiation of neural plate cells induced by Bmp-mediated signals from epidermal ectoderm. Cell.

[35] Tanigaki K, Nogaki F, Takahashi J, Tashiro K, Kurooka H, Honjo T (2001). Notch1 and Notch3 instructively restrict bFGF-responsive multipotent neural progenitor cells to an astroglial fate. Neuron.

[36] Ericson J, Rashbass P, Schedl A, BrennerMorton S, Kawakami A, vanHeyningen V, Jessell TM, Briscoe J (1997). Pax6 controls progenitor cell identity and neuronal fate in response to graded shh signaling. Cell.

[37] Pevny L, Placzek M (2005). SOX genes and neural progenitor identity. Curr Opin Neurobiol.

[38] Hutton SR, Pevny LH (2011). SOX2 expression levels distinguish between neural progenitor populations of the developing dorsal telencephalon. Dev Biol.

[39] Zhang XQ, Huang CT, Chen J, Pankratz MT, Xi JJ, Li J, Yang Y, LaVaute TM, Li XJ, Ayala M, Bondarenko GI, Du ZW, Jin Y, Golos TG, Zhang SC (2010). Pax6 is a human neuroectoderm cell fate determinant. Cell Stem Cell.

[40] Wu JQ, Habegger L, Noisa P, Szekely A, Qiu C, Hutchison S, Raha D, Egholm M, Lin H, Weissman S, Cui W, Gerstein M, Snyder M (2010). Dynamic transcriptomes during neural differentiation of human embryonic stem cells revealed by short, long, and paired-end sequencing. Proc Natl Acad Sci U S A.

[41] Wu H, Xu J, Pang ZP, Ge W, Kim KJ, Blanchi B, Chen C, Sudhof TC, Sun YE (2007). Integrative genomic and functional analyses reveal neuronal subtype differentiation bias in human embryonic stem cell lines. Proc Natl Acad Sci U S A.

[42] Lagger G, O’Carroll D, Rembold M, Khier H, Tischler J, Weitzer G, Schuettengruber B, Hauser C, Brunmeir R, Jenuwein T, Seiser C (2002). Essential function of histone deacetylase 1 in proliferation control and CDK inhibitor repression. EMBO J.

[43] Trivedi CM, Luo Y, Yin Z, Zhang MZ, Zhu WT, Wang T, Floss T, Goettlicher M, Noppinger PR, Wurst W, Ferrari VA, Abrams CS, Gruber PJ, Epstein JA (2007). Hdac2 regulates the cardiac hypertrophic response by modulating Gsk3 beta activity. Nat Med.

[44] Montgomery RL, Hsieh J, Barbosa AC, Richardson JA, Olson EN (2009). Histone deacetylases 1 and 2 control the progression of neural precursors to neurons during brain development. Proc Natl Acad Sci U S A.

[45] Singh N, Gupta M, Trivedi CM, Singh MK, Li L, Epstein JA (2013). Murine craniofacial development requires Hdac3-mediated repression of Msx gene expression. Dev Biol.

[46] Zeng LF, Xiao QZ, Margariti A, Zhang ZY, Zampetaki A, Patel S, Capogrossi MC, Hu YH, Xu QB (2006). HDAC3 is crucial in shear- and VEGF-induced stem cell differentiation toward endothelial cells. J Cell Biol.

[47] Roopra A, Sharling L, Wood IC, Briggs T, Bachfischer U, Paquette AJ, Buckley NJ (2000). Transcriptional repression by neuron-restrictive silencer factor is mediated via the SIN3-histone deacetylase complex. Mol Cell Biol.

[48] Ballas N, Battaglioli E, Atouf F, Andres ME, Chenoweth J, Anderson ME, Burger C, Moniwa M, Davie JR, Bowers WJ, Federoff HJ, Rose DW, Rosenfeld MG, Brehm P, Mandel G (2001). Regulation of neuronal traits by a novel transcriptional complex. Neuron.

[49] Huang HJ, Reed CP, Zhang JS, Shridhar V, Wang L, Smith DI (1999). Carboxypeptidase A3 (CPA3): a novel gene highly induced by histone deacetylase inhibitors during differentiation of prostate epithelial cancer cells. Cancer Res.

[50] Kao HY, Ordentlich P, Koyano-Nakagawa N, Tang Z, Downes M, Kintner CR, Evans RM, Kadesch T (1998). A histone deacetylase corepressor complex regulates the Notch signal transduction pathway. Genes Dev.

[51] Jepsen K, Solum D, Zhou TY, McEvilly RJ, Kim HJ, Glass CK, Hermanson O, Rosenfeld MG (2007). SMRT-mediated repression of an H3K27 demethylase in progression from neural stem cell to neuron. Nature.

[52] Tang Y, Li T, Li J, Yang J, Liu H, Zhang XJ, Le W (2014). Jmjd3 is essential for the epigenetic modulation of microglia phenotypes in the immune pathogenesis of Parkinson’s disease. Cell Death Differ.

[53] Yang D, Li T, Wang Y, Tang Y, Cui H, Tang Y, Zhang X, Chen D, Shen N, Le W (2012). miR-132 regulates the differentiation of dopamine neurons by directly targeting Nurr1 expression. J Cell Sci.

[54] Alev C, Wu YP, Kasukawa T, Jakt LM, Ueda HR, Sheng GJ (2010). Transcriptomic landscape of the primitive streak. Development.

[55] Le W, Yang J, Tang Y: **Expression data of neural progenitor cells differentiation from human embryonic stem cells.** *Gene Expression Omnibus* 2014, http://www.ncbi.nlm.nih.gov/geo/query/acc.cgi?acc=GSE61050.

