# Supplementary figures and images for: Suppression of histone deacetylation promotes the differentiation of human pluripotent stem cells towards neural progenitor cells
Source: BMC Biol. 2014 Nov 19;12:95. doi: 10.1186/s12915-014-0095-z (PMC4254204; doi:10.1186/s12915-014-0095-z)

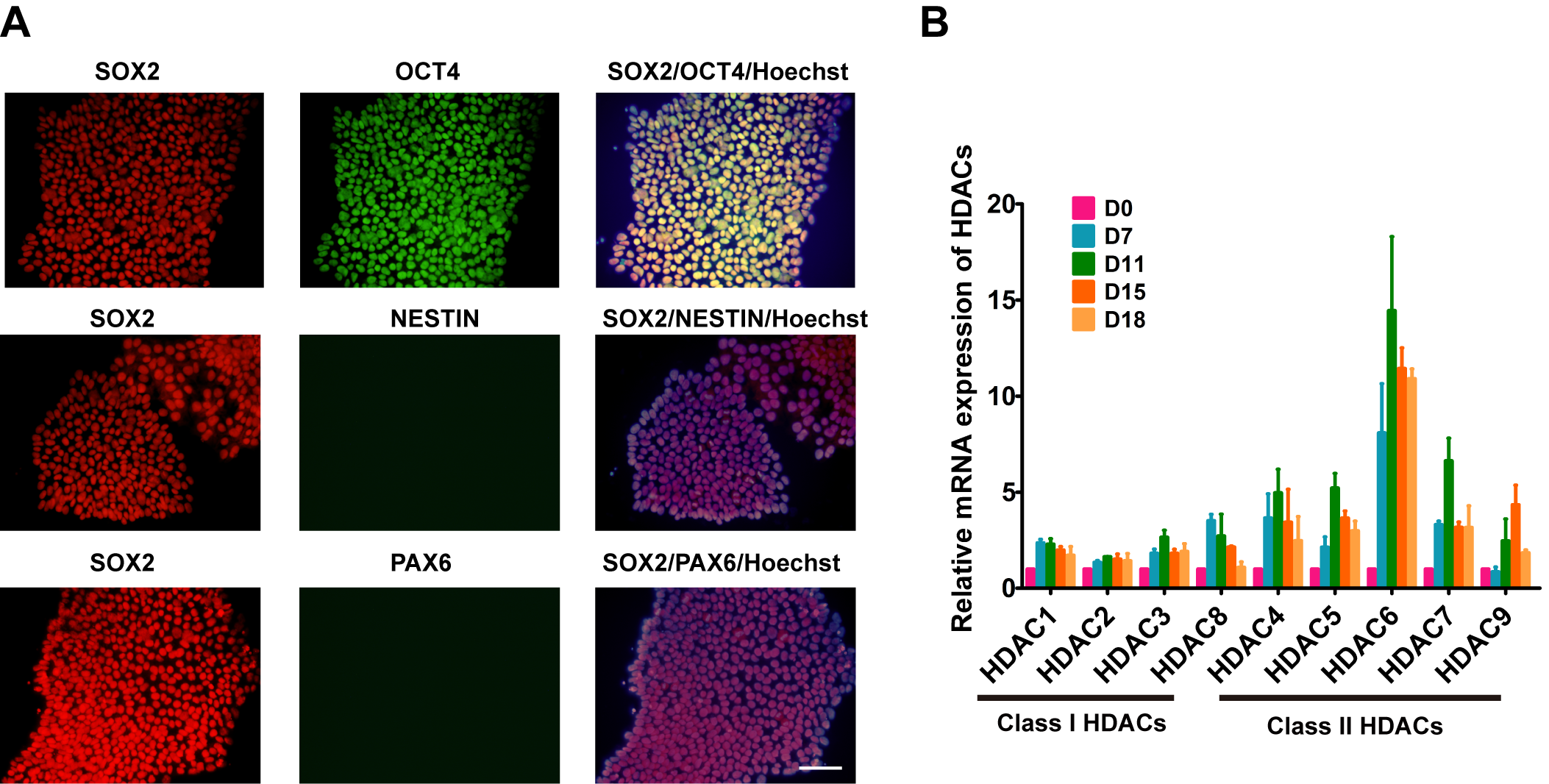

Supplement: Additional file 1: Figure S1 — HDACs participate in the process of neural differentiation. (A) Immunostaining of undifferentiated H9 cells with antibody against OCT4 (green), SOX2 (red), NESTIN (red) and PAX6 (red). Scale bar, 50 μm. (B) The mRNA expressions of class I and class II HDACs during neural differentiation were determined by real-time PCR. The error bars indicate SEM. [file 12915_2014_95_MOESM1_ESM.tiff]

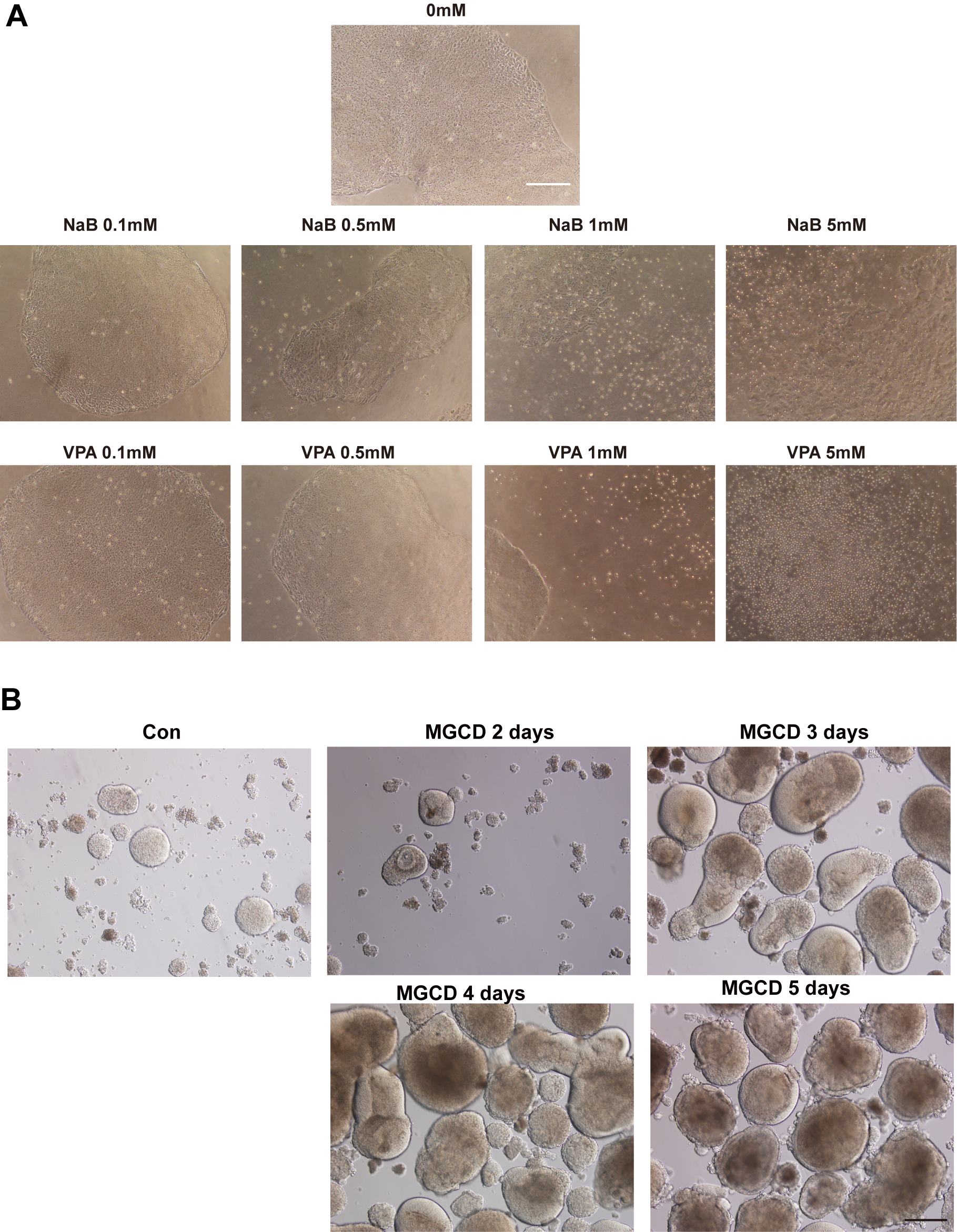

Supplement: Additional file 2: Figure S2 — Concentrations of NaB and VPA and time-dependent manner of MGCD. (A) The morphology of H9 cells was observed after treatment with various concentrations of NaB or VPA. (B) Neurospheres were formed from H9 cells that were induced for neural differentiation with the addition of MGCD for two, three, four and five days, respectively. Scale bar, 200 μm. [file 12915_2014_95_MOESM2_ESM.tiff]

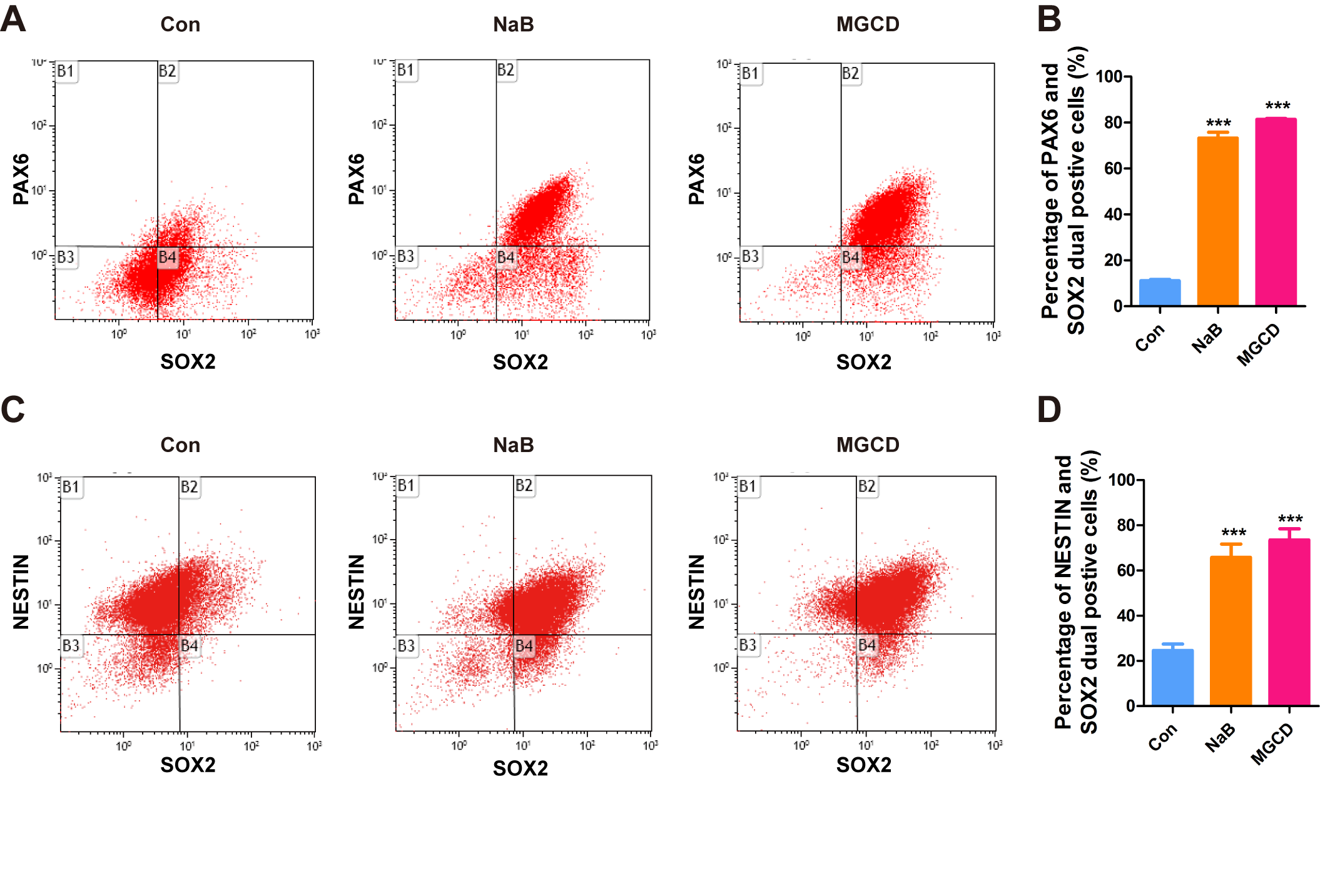

Supplement: Additional file 3: Figure S3 — Flow cytometry analysis of the cells on day 7 of differentiation. (A, B) PAX6 and SOX2 dual positive cells were analyzed and quantified by FC. The error bars indicate SEM, ***P <0.001; n = 3. (C, D) NESTIN and SOX2 dual positive cells were analyzed and quantified by flow cytometry. The error bars indicate SEM, ***P <0.001; n = 4. [file 12915_2014_95_MOESM3_ESM.tiff]

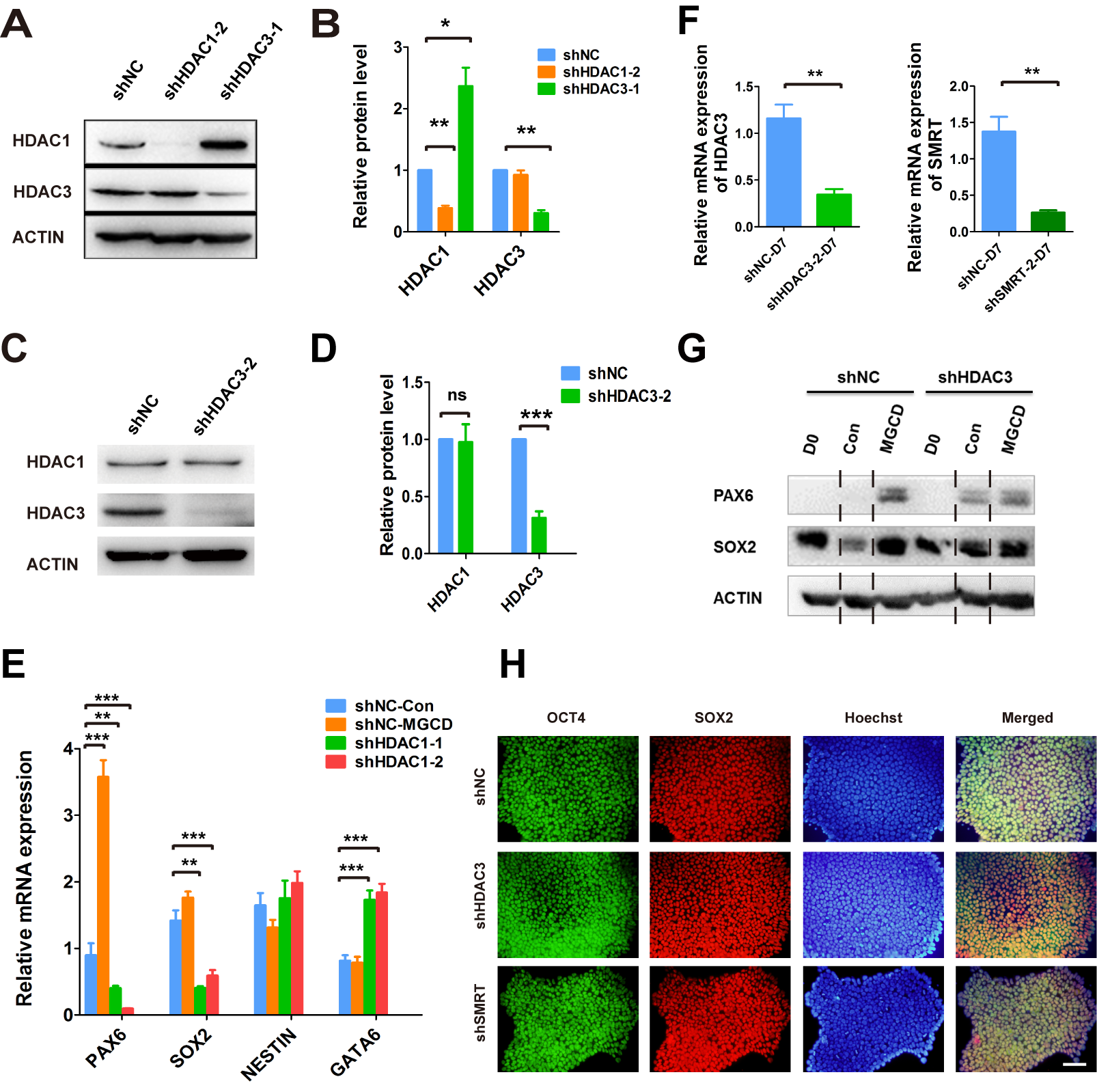

Supplement: Additional file 4: Figure S4 — Neurosphere formation and stemness after HDAC or SMRT suppression. (A-D) Knockdown efficiency of HDAC1 or HDAC3 was determined by western blot. The density of the bands was analyzed using Image J software and expressed as fold of control. (E) The transcripts of PAX6, SOX2, NESTIN and GATA6 in H9-shHDAC1 cells on day 7 of differentiation. (F) Expression of HDAC3 or SMRT in H9-shHDAC3 or H9-shSMRT on day 7 of differentiation. (G) Protein levels of PAX6 and SOX2 were assessed by western blot in shHDAC3 cells with or without MGCD treatment on day 7 of differentiation. (H) Stemness of undifferentiated H9-shHDAC3 or H9-shSMRT cells was detected by immunostaining with anti-OCT4 (green) and anti-SOX2 (red) antibodies. Scale bar, 50 μm. The error bars indicate SEM. *P <0.05; **P <0.01; ***P <0.001; n>/=3. [file 12915_2014_95_MOESM4_ESM.tiff]
