# Supplementary material for: Suppression of histone deacetylation promotes the differentiation of human pluripotent stem cells towards neural progenitor cells
Source: BMC Biol. 2014 Nov 19;12:95. doi: 10.1186/s12915-014-0095-z (PMC4254204; doi:10.1186/s12915-014-0095-z)
Supplement: Additional file 5: Table S1 — A list of primers used for real-time PCR and ChIP analysis. [file 12915_2014_95_MOESM5_ESM.doc]

**Additional file 5: Table S1. A list of primers used for real-time PCR and ChIP analysis.**

| Genes | NCBI accession number | Forward primer |
| --- | --- | --- |
|  |  | Reverse primer |
|  | Real-time PCR Primers | |
| HDAC1 | [NM_004964.2](http://www.ncbi.nlm.nih.gov/entrez/viewer.fcgi?db=nucleotide&id=13128859) | GGGGCCTGCACCATGCAAAGA |
|  |  | TGCCAGCCCCGATATCCCGTA |
| HDAC2 | [NM_001527.3](http://www.ncbi.nlm.nih.gov/entrez/viewer.fcgi?db=nucleotide&id=293336690) | GCCCCATAAAGCCACTGCCGAAG |
|  |  | GCTCCAGCAACTGAACCGCCAG |
| HDAC3 | [NM_003883.3](http://www.ncbi.nlm.nih.gov/entrez/viewer.fcgi?db=nucleotide&id=157266338) | CTGTCTGAACGTGCCCCTGCG |
|  |  | AACGCATTCCCCATGCCCTCG |
| HDAC4 | [NM_006037.3](http://www.ncbi.nlm.nih.gov/entrez/viewer.fcgi?db=nucleotide&id=153085394) | CTGTGACCCAGCCCTCACCG |
|  |  | CACATCCACCGTGCTGGGCAT |
| HDAC5 | [NM_001015053.1](http://www.ncbi.nlm.nih.gov/entrez/viewer.fcgi?db=nucleotide&id=62750348) | GAGTGCCTGACTGCCTCGCC |
|  |  | CGACCTGACATCCCATCTGCCG |
| HDAC6 | [NM_006044.2](http://www.ncbi.nlm.nih.gov/entrez/viewer.fcgi?db=nucleotide&id=13128863) | CTGGCGGAGTGGAAGAACCGC |
|  |  | AGCGGGGAACGGCTCCCTTT |
| HDAC7 | [NM_015401.3](http://www.ncbi.nlm.nih.gov/entrez/viewer.fcgi?db=nucleotide&id=169234806) | ACCCTCGGAGACTCCTCCCCAA |
|  |  | GCCCCAGAGTGATTGCGGGC |
| HDAC8 | [NM_001166418.1](http://www.ncbi.nlm.nih.gov/entrez/viewer.fcgi?db=nucleotide&id=262073087) | GGAACCCTGGCCGAGTCCGA |
|  |  | TACTGGCCCGTTTGGGGATCTTG |
| HDAC9 | NM_058176.2 | GGACGAGAAAGGGCAGTGGCA |
|  |  | GTGGTGGGCAGCCGTGTACC |
| PAX6 | NM_000280.4 | TCTAATCGAAGGGCCAAATG |
|  |  | TGTGAGGGCTGTGTCTGTTC |
| NCAM1 | NM_181351.4 | TCATGTGCATTGCGGTCAAC |
|  |  | ACGATGGGCTCCTTGGACTC |
| HES1 | [NM_005524.3](http://www.ncbi.nlm.nih.gov/entrez/viewer.fcgi?db=nucleotide&id=325652058) | AAGAAAGATAGCTCGCGGCA |
|  |  | TACTTCCCCAGCACACTTGG |
| SOX1 | NM_005986.2 | GCGGAGCTCGTCGCATT |
|  |  | GCGGTAACAACTACAAAAAACTTGTAA |
| HAND1 | NM_004821.2 | TCCCTTTTCCGCTTGCTCTC |
|  |  | CATCGCCTACCTGATGGACG |
| FOXF1 | NM_001451.2 | AAAGGAGCCACGAAGCAAGC |
|  |  | AGGCTGAAGCGAAGGAAGAGG |
| ACTA2 | NM_001613.2 | AAGCAAGTCCTCCAGCGTTC |
|  |  | GCTTCACAGGATTCCCGTCTTA |
| GATA6 | NM_005257.5 | TGTGCGTTCATGGAGAAGATCA |
|  |  | TTTGATAAGAGACCTCATGAACCGACT |
| AFP | NM_001134.2 | AGCAGCTTGGTGGTGGATGA |
|  |  | CCTGAGCTTGGCACAGATCCT |
| SOX17 | NM_022454.3 | TTCGTGTCCAAGCCTGAGATG |
|  |  | GTCGGACACCACCGAGGAA |
| OCT4 | [NM_001173531.2](http://www.ncbi.nlm.nih.gov/nuccore/NM_001173531.2) | CCCCAGGGCCCCATTTTGGTACC |
|  |  | ACCTCAGTTTGAATGCATGGGAGAGC |
| NANOG | [NM_024865.2](http://www.ncbi.nlm.nih.gov/nuccore/NM_024865.2) | GGCTCTGTTTTGCTATATCCCCTAA |
|  |  | CATTACGATGCAGCAAATACGAGA |
| CD133 | [NM_001145847.1](http://www.ncbi.nlm.nih.gov/nuccore/NM_001145847.1) | GTGGCCACCGCTCTAGATAC |
|  |  | TTCAACATCATCGTACACGTCCT |
| SMRT | [NM_001077261.3](http://www.ncbi.nlm.nih.gov/nuccore/NM_001077261.3) | CGCAGTGTAAGAACTTCTACTTCAA |
|  |  | GTTGTTGACAGTGGCTGGGC |
| SOX2 | [NM_003106.3](http://www.ncbi.nlm.nih.gov/nuccore/NM_003106.3) | TTCACATGTCCCAGCACTACCAGA |
|  |  | TCACATGTGTGAGAGGGGCAGTGTGC |
| NESTIN | [NM_006617.1](http://www.ncbi.nlm.nih.gov/nuccore/NM_006617.1) | CGCACCTCAAGATGTCCCTC |
|  |  | CAGCTTGGGGTCCTGAAAGC |
| GAPDH | [NM_002046.5](http://www.ncbi.nlm.nih.gov/nuccore/NM_002046.5) | GGTCTCCTCTGACTTCAACA |
|  |  | GTGAGGGTCTCTCTCTTCCT |
|  | ChIP primers | |
| PAX6-Set a | NM_000280.4 | TCCCCCTCCCCTCTTATCTC |
|  |  | CCCTTTCTTCTCTCCTTTCTCCT |
| PAX6-Set b | NM_000280.4 | GTGTTTTGCTGGAGGATGA |
|  |  | CTGGGAAGGAGACAGAGATT |
| PAX6-Set c | NM_000280.4 | AAAACCCCAACCAAACAAAA |
|  |  | GCAATAAAAATAAAGCGAGAAGA |
